# Parental recommendations for population level interventions to support infant and family dietary choices: a qualitative study from the Growing Up in Wales, Environments for Healthy Living (EHL) study

**DOI:** 10.1186/s12889-015-1561-4

**Published:** 2015-03-11

**Authors:** Ashrafunnesa Khanom, Rebecca A Hill, Kelly Morgan, Frances L Rapport, Ronan A Lyons, Sinead Brophy

**Affiliations:** grid.4827.90000000106588800College of Medicine, Swansea University, Swansea, SA2 8PP Wales United Kingdom

## Abstract

**Background:**

Childhood obesity presents a challenge to public health. This qualitative study explored the main barriers to dietary choices faced by parents with infants, and the types of interventions and policy level recommendations they would like to see put in place, to promote a healthier food environment.

**Methods:**

61 semi-structured interviews with prospective parents and parents of infants (61 mothers and 35 fathers) were conducted. Families were selected according to community deprivation levels using the Townsend Deprivation Index to ensure a representative sample from deprived and affluent neighbourhoods. Inductive thematic analysis was used to analyse the data.

**Results:**

Parents identified triggers which led to unhealthy dietary choices such as reliance on fast food outlets due to; shift work, lack of access to personal transport, inability to cook, their own childhood dietary experiences, peer pressure and familial relationships. Parents who made healthy dietary choices reported learning cooking skills while at university, attending community cooking classes, having access to quality food provided by church and community organisations or access to Healthy Start vouchers. They called for a reduction in supermarket promotion of unhealthy food and improved access to affordable and high-quality fresh produce in the local area and in supermarkets. There was a strong message to policy makers to work with commercial companies (food manufactures) as they have resources to lower costs and target messages at a diverse population. Provision of targeted advice to fathers, minority ethnic parents, and tailored and practical advice and information on how to purchase, prepare, store and cook food was requested, along with community cookery classes and improved school cookery lessons.

**Conclusions:**

There is a need for parent directed community/population level interventions that aims to reduce socio-ecological barriers to making healthy dietary choices. Parents desired improvements in meals provided in workplaces, schools and hospitals, as well as increased access to healthy foods by increasing local healthy food outlets and reducing unhealthy, fast food outlets. Knowledge and skills could then be enhanced in line with these improvements, with confidence gained around cooking and storing food appropriately.

## Background

Maternal and child obesity is a matter of concern in the United Kingdom (UK) with 1 in 5 women of childbearing age (16–44) categorised as obese (body mass index >30 kg/m^2^) [1]. Throughout the UK, 1 in 20 pregnant women have been classed as obese (body mass index >35 kg/m^2^) with a higher proportion found in Wales as 1 in 15 women [2]. Evidence suggests that infants (aged 0–12 months) born to obese mothers are over nourished in the womb, potentially leading to changes in metabolism, behaviour and appetite regulation. These infants are often larger at birth, show increased adipose tissue mass and obesity, and are likely to develop insulin resistance in later life [1,3]. Further data indicates that infant weight gain during the first year of life and even weight status at age 6 months can also have implications for future obesity status, diabetes and other cardiovascular risk factors [4]. Overweight infants are more likely to become overweight children and continue on this trajectory into adulthood [5,6]. Although UK national measurement data on infant weight is not available, data for pre-school aged children suggests an increasing incidence of obesity among young children. The 2010 Health Survey England (HSE) found that 13 percent of English toddlers aged 2–3 years were considered overweight and 10 percent were classed as obese [7]. In Wales the first National Child Measurement Programme (NCMP) published in 2013 found that 22 percent of reception class pupils (4–5 year olds) were found to be overweight or obese [8].

Research evidence indicates that pregnancy presents an opportune time for early prevention of obesity during the life course [9,10]. Infant diet is dependent on choices made by its parents [11,12]. Observational studies suggest that exposure to an ‘obesogenic’ environment often begins in the first 2 years of life. This represents a critical window of opportunity to establish healthy taste preferences that will impact on later health [4,13]. WHO/Europe [14] have restated in a recent report that mothers should aim to exclusively breastfeed their infants until 6 months of age. There is evidence that formula-fed infants consume more calories and are likely to be 2.5 times more obese by age 2 years compared to those who are breastfed for the first 6 months of life [15]. WHO/Europe also recommend that after 6 months of age infants are introduced to a wide variety of foods, including vegetables, to support healthy taste development early on. In the UK the National Institute for Clinical Excellence [10] recommend that mothers maintain a healthy weight before, during and after pregnancy to promote health in their offspring.

Many studies indicate that high consumption of fruit and vegetables by children correlates positively with parental modelling and consumption of fruit and vegetables [16-19]. The National Diet and Nutrition Survey [20] and the Welsh Health Survey [21] found that approximately only a third of adults were meeting the ‘Eat 5 a Day’ recommendation of fruit and vegetable consumption, and indicate that fruit and vegetable consumption is socially patterned with families from the most deprived neighbourhoods consuming less fruit and vegetables compared to those living in affluent neighbourhoods. A closer inspection of the prevalence of maternal and child obesity and overweight data presented above show that childhood obesity also correlates significantly with neighbourhood deprivation. In 2013, European ministers of health recognized the disproportionate impact of obesity on those who are socioeconomically disadvantaged, in the Vienna Declaration [14,22]. They acknowledged the need to: promote access to healthy and affordable food, intervene across the life course, and address gaps in food governance system.

For more than a decade the UK Government has raised concerns about the overconsumption of food high in salt, fat and sugar and the long term negative health consequences for children and health inequality [23]. Attempts to change dietary behaviours have ranged from providing information and healthy lifestyle incentives, to regulatory measures such as the school meal guidelines in England, Wales and Scotland [24-26].

The UK government dietary recommendations [27] advise parents to provide a diet high in complex carbohydrates (whole grains), fruit and vegetables and low fat dairy products, which are more satiating than fat and sugar based products [28]. The Change4life [29] social marketing campaign has been running in the UK since 2009 offering advice to families on how to make small changes to improve their dietary and lifestyle habits. Similarly the government’s Responsibility Deal [30] has initiated voluntary agreements with the food industry to reduce the salt [31] and fat content in food [32]. Recently, campaigners have now added calls for the reduction of sugar found in processed food aimed at children to this list [33]. One study indicates that brands of baby food sold in the UK contain total sugar content higher than 10 percent [34] (government guidelines recommend that no more than 10 percent of average total energy intake should be consumed as sugar) [35]. Critics of the Responsibility Deal have claimed that the policy remains is vague as there are no sanctions or timescales indicating when targets on food reformulation are to be met [36,37].

Government regulatory measures to reduce obesity have included improved food labelling to support consumers to make informed dietary choices at the point of purchase [38] and restricted advertising of unhealthy food products aimed at children on television [39]. However food industry lobbying has led to the failure of the European Union to implement a uniform method of ‘Traffic Light’ labelling system (preferred by consumers [40,41]) across Europe [42], and ‘junk food’ advertisements continue to be viewed by children during family viewing times [43].

The current levels of obesity demonstrate that education on how to make ‘healthy choices’ about food does not necessarily guarantee the adoption of improved dietary habits by the population. Despite the large amount of dietary advice and information found in the media, in schools, on food packaging, in health centres and in doctors’ surgeries, obesity levels remain high. A systematic review of nutritional education interventions aimed at reducing obesity in children less than 2 years of age found limited effect on family diets or parental attitudes towards healthy eating [4]. The review concluded that it was difficult to determine if this lack of improvement was due to small sample sizes, short intervention periods, weak dose effects, or ineffective interventions. Another review [44] to assess the effectiveness of prenatal and postnatal interventions in extending duration of breastfeeding found that long term combined face-to-face information, guidance, and support from a varied range of professionals was most effective. A comprehensive review of recent systematic reviews and meta analyses that looked at interventions to prevent childhood obesity [45] identified that interventions were modestly effective when professional support, education and behaviour change theory was applied concurrently. The review concluded that individual behaviour change interventions would be far more effective if they were developed alongside population-based strategies (to improve the built environment for physical activity; to improve government and industry policy on food quality, availability and pricing; to improve government policy to tackle socioeconomic disadvantage and reduce advertising of unhealthy food).

The theory of reciprocal determinism states that an individual shapes his/her social environment and can also be shaped by it. Thus individual pre-existing behaviours can become entrenched in their social environment and render them resistant to change [46,47]. The failure of health messages to make an impact on rising obesity levels could be related to the poor application of theoretical models or frameworks during the development of such interventions.

Previous research suggests that perception and understanding of risk and the ability to change varies according to socio-demographic factors such as income, education, gender, and ethnicity [48] and the lived environment [49]. Most interventions on obesity have relied on individual-level motivation and education to make good lifestyle choices, rather than identifying ways to improve peoples’ ability to effectively make that change [50]. A study of cancer patients, illustrates that people diagnosed with cancer failed to stop smoking and were unable to change their behaviour to improve their life expectancy, despite being aware of the long-term adverse effects on their health and wellbeing [40].

Current developments in health promotion have acknowledged the importance of broader social and environmental influences on lifestyle choices [49,51-53]. This is reflected in the socio-ecological paradigm that encompasses several different research perspectives, and advocates multilevel interventions both at the individual and community/population-level. The socio-ecological paradigm suggests that the social, community and built environment, along with personal factors are all likely to predict health behaviours [49,53-56]. Multilevel community and population-wide interventions are found to work successfully if programmes are designed to take on a longer-term perspective on health improvement, with the goal to ultimately change the wider social environment so that healthy behaviours can be facilitated within the population [57-60]. Merzel and D’Afflitti [58] conclude that population or community-wide interventions work most effectively when implemented on three levels: 1) individual or family-targeted support that can be offered to high risk populations, 2) community or population-wide social marketing campaigns that can facilitate changes in social norms, and 3) policy-level levers that can be employed to modify the social and political environment, so that people’s ability to make lifestyle changes is enhanced. Parallels can be drawn from the successful implementation of the antismoking ban in the UK which adopted multi-level population wide intervention strategies and has shown year–on-year reductions in the number of people smoking in the UK [61].

In the UK, the Medical Research Council (MRC) provides comprehensive guidance for developing and evaluating complex multilevel interventions. The 2007 updated guidance [62] highlights the importance of understanding local contexts, attitudes, and beliefs using an iterative approach to inform intervention pathways. Similarly, the socio-ecological paradigm advocates that both content-specific and contextual data should be taken into account when informing the development of appropriate interventions [53,55]. A qualitative approach to data collection provides the necessary tool to achieve this iterative process of inquiry. Qualitative research has the potential to contribute to a better understanding of the real-world user perspective (for whom interventions and policies are planned) within the context of wider socio-economic and environmental determinants of health [63]. This kind of formative research is used to inform and design interventions, models or messages with the target audience [64].

There have been many examples of interventions to prevent childhood obesity that have included settings outside the home such as schools, local communities and ‘early year’s’ settings, usually focused on behaviour-change outcomes, for example The Healthy and Sustainable Pre-school Scheme [65]. However Summerbell et al. [66] highlight that there have been few studies which have focused on multi-level intervention for childhood obesity, and to date very few studies have explicitly used the MRC framework for developing and evaluating interventions [67,68]. Furthermore, there are even fewer effective intervention strategies aimed at children under 5 years of age, especially for infants aged 0–3 years [4,69].

This current study used qualitative interviews with parents to elicit evidence on the main barriers and facilitators to dietary choice, and to inform the development of interventions that they would like to see put in place to promote a healthier food environment for their children. We focused specifically on priorities identified by parents at the community/population-level, thus addressing a significant gap in the literature.

## Methods

Participants for this qualitative study were purposively sampled for a diversity of opinions from those participating in an existing birth cohort study “Growing Up in Wales” [70]. The cohort study aims to examine how the environment in which a child lives influences their health in terms of obesity, asthma and injury. Participants were recruited for the ‘Growing Up in Wales’ cohort study when they attended maternity appointments in hospitals and clinics within Abertawe Bro Morgannwg (ABM) University Health Board. Participants who agreed to take part in the cohort study were provided with an information sheet detailing areas of the study. The study protocol and consent forms were approved by the South East Wales Research Ethics Committee for Wales (09/WSE02/37). Recruitment and subsequent research for the birth cohort study and for this current study was conducted according to the ethical principles for medical research set by the Declaration of Helsinki [71]. All participants were aged 16 years and older. They gave their own written informed consent to take part in the ‘Growing up in Wales’ cohort study and again prior to taking part in this qualitative study. Participants were interviewed for this study either during pregnancy or when their child(ren) was/were 12 months old.

Data collection for this present study was conducted according to the guidelines for inductive qualitative research, which includes semi-structured interviews as a means of data collection [72,73]. An inductive approach was chosen as it permits research findings to be influenced by both research objectives and raw data, and provides in-depth rich descriptions. Parental decisions on infant diets are influenced by many factors, therefore we selected face-to-face semi-structured interviewing rather than group data capture methods for this study. Semi-structured interviews offered the opportunity to build rapport [74] with the interviewees, to explore socio-cultural and environmental influences on family dietary choices, thus allow reflection on parents’ described ‘lived experiences’ [75].

A semi-structured interview schedule (see Interview schedule below) was developed underpinned by the socio-ecological paradigm which suggests that the social, community and built environment, and personal factors are likely to predict health behaviours [49,53,54]. The interview topics focused on: (a) parents’ knowledge and views about living a healthy lifestyle and if/how this influenced their current dietary choices for their families; (b) multi-level barriers and facilitators to healthy dietary choices; and (c) population-level interventions and policy changes that parents would like to see put in place to facilitate healthy dietary behaviour for their families. The interview schedule evolved in keeping with the research aims to explore emerging concepts. Interviews took place in the family home (with the exception of two interviews, which were conducted in the workplace).

### Interview schedule

- **Can you describe your diet (individually and as a family)?**
  - *Who does the cooking and shopping in your family? Why/why not?*
  - *Can you describe your meal times (individually and as a family)?*
  - *Can you describe your childhood experience with food?*
- **Does anything pose a barrier to achieving a healthy diet in your family?**
  - *Money, time, etc.?*
  - *Ability to cook or go shopping?*
  - *Location of your home/local shops/supermarkets?*
  - *Beliefs, attitude, culture?*
- **Do you think parents and children need to maintain a healthy diet?**
  - *Can you remember back to when you became aware of the need to eat healthy for the first time?*
  - *What triggered you to make a change?*
  - *Is there anything that would make it easier or harder for you and your family to follow a healthy diet?*
- **Can you identify the best way to inform or advise parents about the importance of healthy eating?**
  - *In an ideal world what sort of help should be available for families?*
  - *What sort of service or support do you look to health professionals for?*
- **Are there other ways you think messages about healthy diets should be given to families?**
  - *Do you think this will make any difference to the health of your family/other families, and why/why not?*
  - *What sort of families do you think will/should use or listen to this advice?*
- **Do you know of anything being done to promote healthy family diets in your local area?**
  - *Are there any initiatives in the area you live in that you have heard about and might want to try out?*
  - *Do you have any ideas for your local area?*
- **Are there any changes you would like to see in your local area that would encourage you and your family to choose a healthy diet?**
- **Is there anything else that can be done to promote healthy diets?**
  - *Would you use the facility/listen to this advice? Why/why not?*
- **Is there anything that would*not*help families to achieve a healthy diet?**

### Data analysis

The analytical process (quotes, codes, categories and themes) followed the principles of inductive data analysis [72] where models and theoretical perspectives are informed by the interpretation of raw data. Interview transcripts were anonymised and transcribed verbatim to produce transcripts of narrative text for inductive thematic analysis by three researchers (AK, RAH and SB). The researchers worked independently to systematically draw out themes and categories from the interviews based on valid inferences on potential barriers and facilitators and on parents’ ability to make ‘healthy choices’ in the environment they live in. The first 20 transcripts were open-coded to identify quotes which could illustrate emerging concepts in relation to these categories. These open-codes and linked quotes then formed the basis of a codebook that was used to analyse the remaining transcripts to reduce interpretation bias. Throughout the coding process researchers discussed the code-book to ensure inter-coder reliability and coding consistency, and emerging categories were examined for convergences and divergences in the data. Findings were discussed through four paired analysis sessions, to ensure inter-coder correlation [72,76]. Once the coding process was complete, codes were refined and clustered to allow sub-categories to be revealed. To ensure veracity of method, a senior qualitative researcher (FLR) was available during the analysis process to challenge and critique analytic outputs and themes as they emerged.

Data collection was discontinued once researchers, who worked together to clarify themes, concluded that no new themes were emerging (following thematic analysis of interview data in batches of 20 interviews). Theoretical saturation [77] was reached when 40 transcripts had been analysed with the remaining 21 adding detail to the thematic findings.

## Results

A total of 61 interviews were conducted by a trained researcher (AK) with parents over a period of four months (see Table 1). The average time for each interview was approximately 30 minutes and interviews were recorded and transcribed verbatim and checked against audio-recordings. Four interviews were conducted in the participant’s mother language (Bengali (3) and Urdu (1)) and were translated and transcribed by the researcher (AK). There were 61 mothers in the study and their ages ranged from 20 to 42 years. Thirty five fathers, aged between 21 to 52 years, were also interviewed along with the mothers. The average age for mothers was 30 years and for fathers, 35 years. The final interview sample included 26 families from affluent neighbourhoods and 35 from deprived neighbourhoods. Families were categorised into these two groups according to the Townsend Deprivation Index [78], comprised from Census-based data. Four variables were used to score this index: households without a car, overcrowded households, households not owner-occupied and the number of persons unemployed within the household. Further family characteristics can be found in Table 1.

**Table 1:** **Demographic characteristics of participants**

**Characteristics (n)**		
**Number of interviews conducted**		**61**
**Gender (n)**		
Mother		61
Father		35
	**Mother**	**Father**
**Average age in years (age range of all parents)**	30 (20–42)	35 (21–52)
**Occupation of all parents (n)**		
Non manual	16	27
Manual	16	26
Student	5	3
Not in employment	11	4
Homemaker	12	1
**Education of all parents (n)**		
Higher degree	16	12
First degree	15	13
Diplomas in higher education	6	3
A/AS levels	4	3
O/GCSE levels	14	14
Other	2	12
None	4	3
Unknown	0	1
**Ethnicity of all parents (n)**		
White (European)	**50**	**52**
Welsh	48	51
Romanian	1	0
Polish	1	1
Ethnic minority	**11**	**9**
Bangladeshi	4	4
African	2	1
Middle Eastern	2	2
South East Asian	1	1
Pakistani	2	1
**Socio-economic status % (n)**		
Affluent neighbourhood	43% (26)	
Deprived neighbourhood	57% (35)	
**Number of children % (n)**		
Expectant mother with no child	11% (7)	
Expectant mother > 1 child	48% (29)	
Families with child ≤ 12 months of age	41% (25)	
Average number of children per family (range(n))	1.7 (1–5)	
Average age of children (age range)	4.75 years (1 month-18 years)	
**Breastfeeding (children ≤ 12 months of age) % (n)**		
Plan to/have breast fed child	41% (25)	
Plan to/have breast fed and bottle fed child	36% (22)	
Plan to/bottle fed child	23% (14)	

Completion of data analysis produced three key themes: ‘community’, ‘family and individual’, and ‘policy-level influences’ on dietary choices. Finally recommendations from parents on ways to improve dietary choices for young families were sought.

### Community level influences

Community and culture were strong factors in determining infants diet as it was deemed easier and preferable for parents to continue cultural taste preferences and habits that their communities held and reinforced:

*…it’s not healthy actually its oily, it’s our traditional food that we have grown up with…we are not trying to eat healthy. We are just following the past generations…we are just carrying on not thinking about our health (deprived neighbourhood-mother 4: Child aged 4 months).*

In many lower-income families, there was the perception that unhealthy food was an affordable treat that could be enjoyed by the whole family. Parents felt that they needed to provide the best for their children and were perturbed at that the thought that their peers would view their child as deprived:

*…it’s hard when you haven’t got much money and they want ice cream and they want chips…Don’t get me wrong, I do get my little boy McDonalds, don’t think I’m nasty (deprived neighbourhood-mother 14: Children aged 6 years and 9 months).*

There was a perception among some families from less affluent areas that some factors affecting their health were simply out of their control, fostering low expectations and a fatalistic outlook concerning their health.

*If you are going to have cancer you are going to have it, there is no way of stopping it…. I was going to try Weightwatchers but, you know, there’s bigger people out there than me I think then so why should I worry? (deprived neighbourhood–mother 61: Children aged 1 year and child aged 10 months).*

*I don’t know really [what we would want to see improved in the local area] because he [husband] grew up here as well and it’s like we’re kind of used to it. We were used to not having loads of stuff [local facilities] (deprived neighbourhood–mother 40: Child aged 9 months).*

*I don’t eat as good as I should. But like I said as long as X [daughter] does I don’t care about me (deprived neighbourhood mother 15– Child aged 3 years and 1 month).*

The level of deprivation and structural inequality within the community was a contributory factor to dietary choice, with financial barriers cited as the major reason why families from deprived neighbourhoods were unlikely to buy fresh food. They preferred to purchase frozen food or jars of food that would keep longer, reduce waste and were less costly. Meal skipping in such circumstances occurred as a practical response to income inequality, with many families in deprived communities only having one meal a day [79]. Among the interviewees, there were parents who regularly went without meals, compromising their nutritional needs in order to make sure their children were provided for:

*We don’t eat breakfast…X(child) has breakfast. We go without breakfast… it’s just another cost to us, isn’t it? (deprived neighbourhood – father 21:Child aged 4 years and expecting child).*

Some mentioned that skipping meals was an eating behaviour learnt from family. This behaviour could indicate a form of adjustment to poverty, learnt over time [80]:

*Well I go about days without food, it doesn’t bother me like…Yeah, me and my mum’s the same, we just…We don’t eat actually, we have the odd meal here and there…(deprived neighbourhood – father 29: Child aged 1 year and expecting child).*

Others mentioned that they ate if they were in the mood to eat, which could be associated with symptoms of depression [81] related to low socio-economic status.

*I’ve always been the same, I only ever eat when I’m hungry but then I eat what I want to eat…(deprived neighbourhood – mother 18: Child aged 3 years, and child aged 9 months and expecting child).*

Parents cited being ‘busy’ (due to shift work, caring for children, and activities outside the home) as another reason for unstructured meals. They were unable to invest in the time required to prepare food:

*as for time to sit down and have a set meal, don’t always get it so…[wife] had the kids all day, she’d go to work and then I’ve got them for about two hours …[with] the recession…money is very important to everybody so taking time off work might be detrimental to looking after the kids (deprived neighbourhood–father 60: Child aged 1 year and child aged 8 months).*

*I’ve learned a few things, but they take too long… you can’t be chopping up apples, there is no time when the baby’s pulling on my legs to get up… so that’s kind of gone out the window now (affluent neighbourhood–mother 31: Child aged 1 year and expecting child).*

In such circumstances the culture of fast food, where instant food is available (cooked in the microwave or oven), perpetuated behaviours, where meals are not planned, and food is just ‘grabbed’ when passing or just taken when really hungry.

However some families did take pride in providing healthy food for their children, even if they could not afford it for themselves. These were families who had attended community-centre cookery classes, or who came from families that had always cooked. Taking pride and having the confidence to cook healthy food from basic ingredients was more common amongst those who had a university education, as the following quote indicates:

*Living with students and what have you, so I’ve picked up skills (affluent neighbourhood-father 25: Child aged 2 years and expecting child).*

Importantly, accessibility to fresh food varied between local areas. For some families in deprived areas limited access to personal transport in addition to there being no local shops within walking distance became a major barrier to the purchase of fresh food. These families became reliant on takeaways as a result of difficulty in accessing supermarkets, and carrying bulky foods items home on a child’s buggy made buying a large weekly shop impossible. However some areas did offer local initiatives that sold affordable fruit and vegetables and delivered to the door, or had church-run facilities which offered subsidised healthy meals for families:

*I can’t get everything on the buggy on my own so we do go without, we eat terribly [rely on takeaways] (deprived neighbourhood-mother 16: Child aged 9 months).*

*We are quite lucky…because a van comes round once a week with fresh fruit and veg (deprived neighbourhood-mother 27: Children aged 10, 11 years and expecting child).*

### Individual and family barriers and facilitators

The findings indicate that some families who had access to good quality fresh foods chose not to purchase them, because they lacked cooking skills, as they had been brought up by parents who had not cooked at home. Some parents worked shifts, so wanted quick food, or felt that they did not want to take the time to prepare and to clean up after cooking [82]. In contrast, there were families who had limited access to fresh food but who made a determined effort to provide healthy food for their children:

*I think it is cheaper to go and buy fresh and cook it yourself than ordering food or buying food from outside, if you look at a pizza you order, £15, that £15 you can cook a lot of food which is going to be healthier, 100 percent healthier (deprived neighbourhood-mother 13: Children aged 8 years and child aged 8 months).*

Parents described how families strongly influenced each other’s dietary behaviours. If one parent was found to be motivated towards living a healthy lifestyle then this influenced the rest of the family. Notably when parents separated or entered new relationships, the current family diet could either change to become healthier or less healthy, depending on the partners preference, illustrating that diets can change:

*Before he (partner) met me he would live on ‘McDonald’s’, he was really bad …These days he’s [eating well]…he’s lost a lot of weight and he’s looking a lot better (deprived neighbourhood - mother 19: Child aged 9 months).*

Personal preferences and personality of the child influenced what they were given to eat. Parents reported that children may be happy to eat healthy food with others but wanted less healthy meals at home. If children continually refused healthy food, parents often provided unhealthy food that they knew the child would eat [13].

*Yeah chicken nuggets, it is a must for him, every day, every dinner, lunch, because that is only what he wants to eat (affluent neighbourhood - mother 59: Child aged 5 years and expecting child).*

In addition, some parents were aware that their diet was unhealthy and that their child might mirror their unhealthy dietary behaviours, so they ate out of sight of their child, ate when the child was in bed, or just would not let their child eat the same food as them:

*The kids always have their meals…but then he’ll perform for mine and I can’t really give it to him because it is full of salt and that (deprived neighbourhood - mother 14: Children aged 6 years and child aged 9 months).*

Importantly, we identified that when parents approached major milestones in their life, or during times of change, they thought about their diet and their family’s diet. For some, becoming pregnant, turning 30 or 40 years of age, or having an older family member diagnosed with a health problem, were times when they were more open to making positive lifestyle changes, like giving up smoking, or making the choice to start eating more healthily.

### Policy level influences, barriers and facilitators

Findings from the study suggest that parents were aware of dietary messages encouraging them to eat more fruit and vegetables, particularly the ‘Eat 5 a Day’ message, but no parent mentioned the social marketing initiative Change4Life, championed by the government at national and community-level. The initiative may have failed to capture the public imagination and may need to be publicised widely and in different ways.

Many parents were unsure about the kind of diet they should be providing their infants and how to achieve this in a practical way. They found health visitors unhelpful and believed they gave prescriptive messages:

*Perhaps they [health visitors] are knowledgeable, perhaps they just don’t have the time to give…when I was first weaning X [son] you know like what proportions of meat and veg I should be giving him…that’s not something that I know and they were quite unhelpful to be honest (deprived neighbourhood - mother 17: Child aged 1 year).*

Parents commented that supermarkets encouraged unhealthy diets by advertising and promoting unhealthy food and not targeting the promotion of fruit and vegetables:

*A few weeks ago we was in X [supermarket] and they were giving out free ‘Haribos’ to let you try it … that’s not particularly helpful for parents. (Laughs)… They never give out a free banana (deprived neighbourhood - mother 17: Child aged 1 year).*

Parents suggested that fruit and vegetable packaging could carry recipes to encourage home cooking.

However, parents in deprived neighbourhoods praised the provision of government ‘Healthy Start’ vouchers for 0 to 4 year-olds, which afforded them the opportunity to purchase healthy fruit and vegetables for their young family:

*‘Healthy Start’ vouchers…I think they are a good thing because you can only buy milk and fruit and veg, so they’re really encouraging (deprived neighbourhood - mother 16: Child aged 9 months).*

### Recommendations

#### Cost and accessibility

Parents felt that supermarkets should increase affordably priced, high-quality healthy food, and reduce promotions on unhealthy food. Parents called for the provision of more healthy ready meals and takeaways, and more promotions on healthy foods in supermarkets. There was a great deal of support for ‘Healthy Start’ vouchers to buy fruit and vegetables as they were seen as vital in improving the family diet. It was felt that this was a good initiative that should be continued and that the threshold for receiving these vouchers should be raised, as some families on low income fell outside the eligibility criteria.

Parents also recommend that existing public facilities should be used more effectively. They suggest that public transport should be made cheaper to enable families to access better-quality produce further away from home. There should also be more emphasis in the school environment to promote healthy dietary behaviour, during and after school time, and more direct engagement between parents and schools. It was suggested that cooking lessons in schools for children could be improved, and parents could be invited to food tasting events, cookery classes and afterschool clubs that provided good dietary advice to children and parents. Furthermore, it was proposed that work places could be used to provide information about personal health status, information on healthy lifestyles, and how to make lifestyle changes:

*There’s the market in the Swansea [sells cheaper fruit and vegetables] but you’d have to drive… the bus is actually quite expensive…(affluent neighbourhood - mother 35; Child aged 4 years and expecting child).*

*When we mapped our own diets and lifestyles* [in the workplace health awareness class] *we realised that we needed to make changes (affluent neighbourhood - father 49; Child aged 4 years and 1).*

#### Improved information

Parents advised that information on healthy eating should be practical, easy to adhere to, and incorporated into everyday living. It was felt that people should be made aware of what was actually healthy. Advice provided should be tailored specifically towards different groups, for example to target written information and advice in a way that was more applicable to fathers or to provide face-to-face information to minority ethnic families, would make it easier for different constituencies of the population to connect with healthy lifestyle messages:

*I think [it would be good] if someone’s giving out like good information for dads [on healthy eating]…(affluent neighbourhood - father 52: Children aged 10 and 9 years and expecting child).*

#### Legislation

It was felt that the government should ensure that food manufacturers produce food low in salt, sugar and fat content, that food labelling should be clearer, and that there should be legislation in place to improve the quality of food. Importantly, parents wanted food provision in schools, hospitals, workplaces and other institutions to model healthy food choices. It was suggested that food procurement departments should avoid opting for the cheapest commercial food provider:

*I spent a lot of my time in the hospital because of my problem, and when I was pregnant I had to basically bring my own food because they don’t give you fruit and veg very much, they don’t do fruit unless it’s things like apple crumble…(deprived neighbourhood - mother 33: Child aged 2 years and expecting child).*

O*n Monday they [children] had pizza and chips [for school lunches]. I was quite devastated at that (affluent neighbourhood - mother 48: Children aged 18, 10, 4, 1 years).*

Parents recommended that ‘fast food’ outlets should be restricted, and that the way neighbourhoods were planned and built should facilitate access to local, healthy food. It was also suggested that the government should subsidise cheaper healthier food:

*There is nothing to promote healthy living [in the local area] … you’ve got a chip shop, a kebab house (deprived neighbourhood - mother 21: Child aged 4 years and expecting child).*

It was widely felt that the government needed to play a more active role in improving work-life balance as shift work was a major influence on families who had adopted unhealthy dietary behaviours:

*I’m working and my job is different hours virtually every day so I could be having lunch at half past ten one day and then it might be half past 12 the next day… I don’t know if the government could do anything…[so] we could spend more time with the kids (deprived neighbourhood - father 60: Child aged 1 year and 8 months).*

#### Media

Television was considered an important source of information and inspired some parents to follow a healthy lifestyle, in particular scientifically-supported programmes on food and diet appealed to fathers. Programmes which made parents think about their offspring’s future physical appearance, or how unhealthy food could affect the body in a harmful way, or offered advice on how to feed young children and described a healthy diet for a child, were greatly valued. Programmes that presented good role models to children, such as ‘Sporticus’ in ‘Lazy Town’, were praised, as were TV chefs who advocated healthy eating or encouraged people to cook, such as Jamie Oliver:

*I’m actually watching a programme on TV about healthy eating now. It’s always interesting, we find out about certain foods and how they help you (deprived neighbourhood - father 27: Children aged 10 and 11 years and expecting child).*

## Discussion

This study examined factors influencing infant diet and included parents’ recommendations on ways to improve family and infant dietary choices. Parents who participated in the study understood the pre-requisites of a healthy diet, and information such as ‘Eat 5 a Day’ [29] or the ‘Traffic Light’ system of food consumption, but were often unsure how to make key changes in their own lives. There were various barriers to providing a healthy family diet, these included: the ready availability of fast food; difficulty accessing fresh fruit and vegetables; peer pressure from others in the community to conform to typical diets, or to not deny the family affordable (unhealthy) treats.

There was a fatalistic, or apathetic approach to the long term health consequences of poor dietary choices among some families living in deprived neighbourhoods. Parents’ lives were evaluated in the context of the environment they lived in [83] and the dangers associated with a diet high in fat, salt and sugar were considered a less risky lifestyle choice, compared to living in a deprived neighbourhood.

The findings highlight the significance of past and present dietary experiences of parents. Parents’ own childhood food experiences, their own parents’ ability to cook, the parents’ individual food preferences, and their personal views on the best way to feed children influenced the food choices they made for their infants.

Interestingly we found that 77 percent of mothers in this study hoped to or had initiated breastfeeding. The data is comparable to Welsh national breastfeeding data from 2010 which shows breastfeeding initiation rates at 71 percent. Welsh data also indicates that 23 percent of mothers were breastfeeding their children up to the age of 6 months. In our study 41 percent (n = 25) of mothers planned to or were exclusively breastfeeding their infants. Whilst 36 percent (n = 22) chose to breast and bottle feed and 23 percent (n = 23) had decided to just bottle feed. Only one mother (from an affluent neighbourhood) in this study had commented that there was too much pressure placed on women to breastfeed. She chose to forego breastfeeding as it would be more convenient for her when she returned to work, as a teacher after the birth of her baby.

The Welsh government has had a breastfeeding strategy in place since 2001 [84] and has supported local breastfeeding initiatives which complement its strategy to reduce obesity levels in Wales. The UK government also has policies in place to support women who return to work and would like to continue to breastfeed [85]. Whilst policies and strategies are in place, widespread sociocultural barriers to breastfeeding remain [86].

The main policy level recommendations from parents focused on the accessibility and affordability of good quality, healthy food: they wanted improved ease of access to low cost healthy foods (in their local area and further afield), reduced promotion of unhealthy food (in their local area and in supermarkets), provision of targeted advice to fathers and minority ethnic parents, and tailored and practical advice and information on how to manage time, purchase, prepare, store and cook food for themselves and their children. There was a strong message to policy makers to work with commercial companies (food manufactures) as they have the necessary skills and resources to lower costs, and target messages at different groups of people. Parents were of the opinion that food providers should be subject to legislation, to improve the quality of their food and they should restrict access to and the sale of unhealthy food. Similar recommendations were made for healthy food provision in public facilities such as hospitals, workplaces and schools.

Opinions expressed by parents’ in this study are supported by existing literature. It is recognised that family food choices are influenced by complex factors outside their control. Cultural beliefs, values and local availability of healthy food have been identified as barriers by many others [87-89]. Furthermore, family income, convenience, and food preferences of family members [13,16] such as the father [90], are additional barriers to ‘healthy choices’.

Research indicates that low cost, energy dense, and low nutrient foods high in salt, sugar and fat, are consumed largely by low income families [91,92], highlighting a link between poor dietary choices and deprivation. These families have adapted to structural inequality by developing irregular meal consumption patterns [79]. There was a perception among interviewees in this study that healthy food costs more and this coincided with the general low consumption of fruit and vegetables described overall [13,19]. Supporters of healthy eating have called on the government to do more. They have suggested greater regulation of the food industry, clearer labelling and introduction of taxes on unhealthy food [93,94]. The parents in our study made similar requests and desired the provision of ‘good quality’, fresh produce at affordable prices [95]. They questioned why processed food was cheaper than fresh produce, but on the question of taxing unhealthy food they held mixed views [96].

It is well documented in the literature that an over-abundance of fast food outlets as opposed to local fruit and vegetable retailers in an area is conducive to an ‘obesogenic environment’ [97-100], that increases the opportunity to consume unhealthy food. We found that not only are there macro obesogenic environments but respondents also suggested that micro-obesogenic environments can be found in supermarkets. In-store marketing of unhealthy food and promotional offers on processed food encouraged unplanned meals and over-consumption [101]. Providing recipes on packages of fresh produce could be a possible way to mitigate against the effects of such environments as suggested by parents.

It has been argued that in an obesogenic environment the degree of influence parents can have on their growing child’s diet is questionable [102]. In fact, the social pressure on parents to provide fast food to their child echoes Bourdieu’s theory of ‘cultural capital’ [103], where similarities in taste and dietary behaviour is perpetuated by the community to create a sense of collective identity. This was most prevalent among families from deprived neighbourhoods. Consuming fast food was seen as a treat [104] and had taken on a “…‘market value’ in the struggle for privilege…” [105] that became an alternative way to achieve a designated status in the community and practised as normalised behaviour [80,106]. In contrast parents living in affluent areas mostly talked about the importance of eating healthy meals at home and outside the home, and negated the consumption of unhealthy processed or fast food.

Research has shown that infant dietary taste and preferences start to develop in the womb and continue to develop in early infancy (encouraged by breast milk consumption) [13,107,108]. Good dietary habits are developed in the first three years of a child’s life [109,110]. Our findings did not completely support this viewpoint, because we found dietary habits were flexible and greatly influenced by changing family circumstances and by life experiences (e.g. learning to cook, healthy peers promoting a healthy diet), highlighting the social influences on diet [111].

The findings here indicate that parental education and professional employment protect infants from an unhealthy diet [112]. Parents in our study who had had the opportunity of a university education (including those who had been bought up in deprived neighbourhoods) had more exposure to new food and improved or developed their cooking skills as a consequence of living independently. The ability to cook meals from fresh ingredients influenced dietary choices and this is supported by the literature [113,114]. However, parents were critical about cookery lessons they had received in school. They asked for improved cookery lessons in school, sought tailored advice aimed specifically at fathers, or ethnic minority families, or advice as to how they could change their socio-economic circumstances for the better. Marks et al. [115] have argued that health-orientated messages need to be simple, clear and consistent to be effective. Others have suggested that cogeneration of knowledge [116] regarding risk to health is required between policy makers and the target community/population, to increase adherence to health messages and prevent ill health.

### Study strengths and limitations

This study complies with the RATS guidelines for reporting qualitative research [117]. Participants in this study had already agreed to participate in a birth cohort study and so as such may have been more open to change and motivated towards healthy behaviours compared to participants who had not already been recruited into a birth cohort study. However, we purposively recruited fathers, minority ethnic families and families from deprived neighbourhoods, to add a counter-balance to some extent. This enabled us to elicit diverse opinions on experiences and beliefs that influence the quality of infant diets. A number of priorities for community/population-level intervention were identified, but may not be generalizable to the wider population as research was conducted in a small area, with a limited sample size. Although data saturation was achieved, it would be difficult to assess if the resultant findings are universal to all parents. However given the need to reduce population wide obesity by preventing obesity from infancy onwards, it warrants further investigation by policy makers into the feasibility of implementing community/population-level interventions recommended by parents. Further research should carry out direct observations to ascertain actual practices rather than parent reported practices of family dietary experiences in the community and this would help to further understand the complex barriers to healthy eating, in order to inform multi-level policy and intervention development.

## Conclusions

Parental recommendations for improving infant diets focused on reducing barriers to accessing high-quality, low cost, healthy foods in local communities, workplaces and schools. Legislation was recommended to reduce access and affordability of unhealthy foods and to improve the obesogenic retail environment and family work-life balance. Recent policy initiatives complement some of the recommendations made by parents such as the mandatory guidelines in Wales and Scotland aimed at improving hospital food [118,119] and school meals in England, Wales and Scotland [24-26].

To enact the recommendations suggested by parents in this study will require health professionals, local and national policy makers, the media and the food industry to engage with both mothers and fathers, to challenge determinants of inequality in dietary intake, and to tailor information towards those most in need. In the current food environment, untailored support and advice was found to be particularly unsuitable for families in deprived neighbourhoods, who had a tendency to embrace a more fatalistic attitude towards their family’s health and wellbeing. They saw many aspects of their lives out of their control and understanding health risks associated with a poor diet was seen as less of a priority. Parents would benefit from a participatory approach to policy making that can create local environments that reduce inequality, meets their needs and supports them to make healthy dietary choices as recommended by the recent NHS England report ‘Five Year Forward View’ [120].
